# Surgical Repair of the Postoperative Lung Hernia by Combining Mesh Interposition and Chest Wall Stabilization by Using Synthes Plates: A Novel Technique

**DOI:** 10.1155/2019/2107083

**Published:** 2019-04-07

**Authors:** Dragan Subotic, Mark Wiese, Aljaz Hojski, Didier Lardinois

**Affiliations:** Clinic for Thoracic Surgery, University Hospital Basel, Switzerland

## Abstract

Several technical points for postoperative lung hernia repair are still not fully elucidated. We present an original technical solution to deal with this complication. In a 68-year-old female, the lung hernia was confirmed 5 months after the partial left-sided chest wall and scapula angle resection with primary Mersilene mesh reconstruction for elastofibroma. The patient refused the proposed surgical correction, being only slightly limited in daily activities. The symptoms persisted under analgetic therapy till the moment when patient's daily activities became critically limited, 22 months after surgery. The repeated chest CT showed a slight increase in hernia size with no signs of tumour recurrence, so that reoperation was planned. After the exposure of the mesh region, a lung protrusion (4 × 3 cm) along the anterolateral edge of the mesh was confirmed. By careful dissection, the mesh was separated from a firmly adherent lung and removed. After adhaesiolysis and complete lung liberation, a wedge resection of the afunctional lung tissue of the lingula was done, just in the region of contact with the mesh. After the chest tube insertion, the chest wall defect was reconstructed by using a Mersilene mesh, and the final chest wall stabilization was done by the fixation of two Synthes plates (DePuy Synthes J&J) over the 5th and 6th ribs. The postoperative course was uneventful. One year after the operation, the patient was in good general condition, without the need for analgesics. To the best of our knowledge, the described technique is the original way of dealing with postoperative lung hernia. We find it efficient as a prevention of potential serious hernia-related complications.

## 1. Introduction

Lung herniation is a protrusion of the pleural-covered lung parenchyma beyond the normal boundaries of the pleural cavity [1], with no more than 400 cases reported till 2013 [2]. Despite sufficient data about the indications for surgical repair, several technical points for particular hernia types are still not fully elucidated. Although technological advent enabled the use of novel materials aimed at improving postsurgical chest wall stability, like titanium implants, literature data are still scarce.

We present a novel technique of the postoperative lung hernia repair, by combining the mesh interposition and Synthes plates. To the best of our knowledge, there is only one paper published so far dealing with lung hernia in a similar way.

## 2. Case Presentation

**Figure 1:**
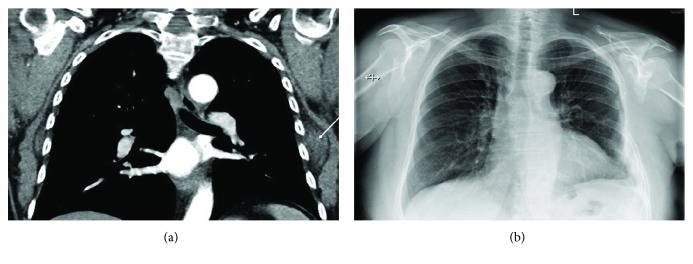
Chest radiography before and after the first operation. (a) Before the operation; arrow: the chest wall tumour. (b) At discharge.

**Figure 2:**
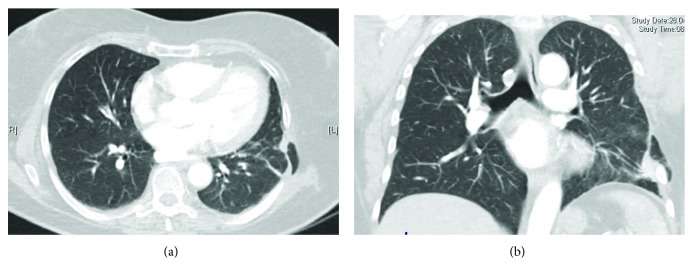
Computerised tomography of the chest at the time of hernia diagnosis. (a) Axial view. (b) Coronal view.

**Figure 3:**
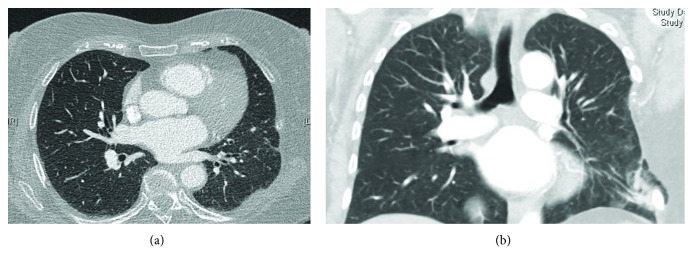
Computerised tomography of the chest at the time of hernia repair.

A 68-year-old female underwent a partial left-sided chest wall resection, with partial removal of the 6th and 7th ribs and of the scapula angle for elastofibroma (Figure 1(a)). The chest wall defect was reconstructed by using a Mersilene mesh, secured by interrupted pericostal stitches, and covered by a sufficient volume of viable muscles. The postoperative course was uneventful; the radiographic aspect at discharge was normal (Figure 1(b)). The first symptoms in the form of pains in the region of the incision appeared five months after the operation, and computer tomography (CT) of the thorax showed a lung hernia in the region of the mesh covering the chest wall defect (Figures 2(a) and 2(b)). The patient refused the proposed surgical correction, being only slightly limited in usual daily activities. During the next several months, the symptoms persisted with variable intensity under analgesic therapy, till the moment when pains significantly limited patient's daily activities, 22 months after the operation. The repeated chest CT showed a slight increase in hernia size, with no signs of tumour recurrence (Figure 3), so that reoperation was planned.

**Figure 4:**
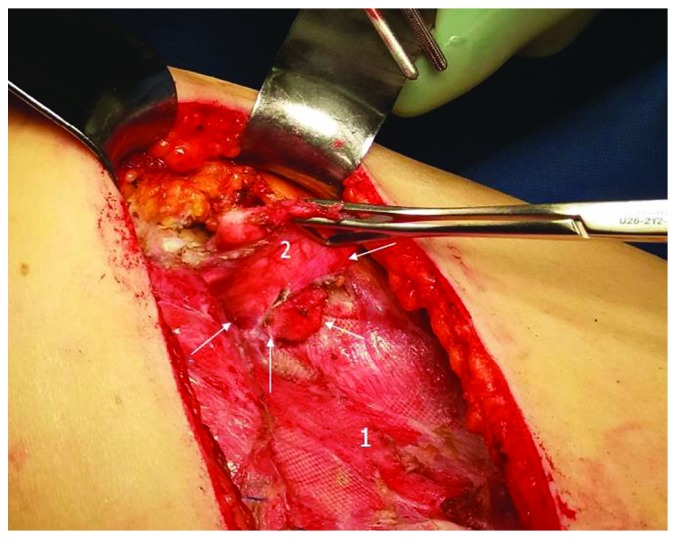
Operative view after hernia exposure. Arrows: mesh disruption region; 1: mesh; 2: lung hernia.

**Figure 5:**
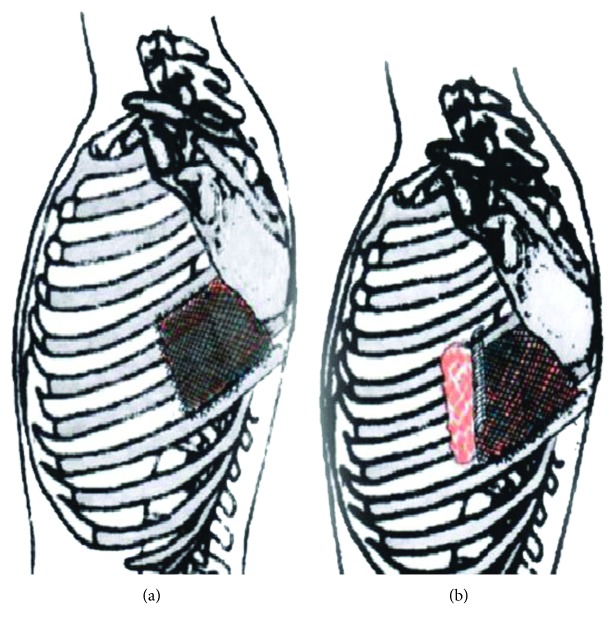
Schema of the local situation at the first and redo surgeries. (a) First surgery: mesh in place over the chest wall defect; scapula angle removed. (b) Redo surgery for hernia repair: lung protrusion along the disrupted anterolateral part of the mesh.

**Figure 6:**
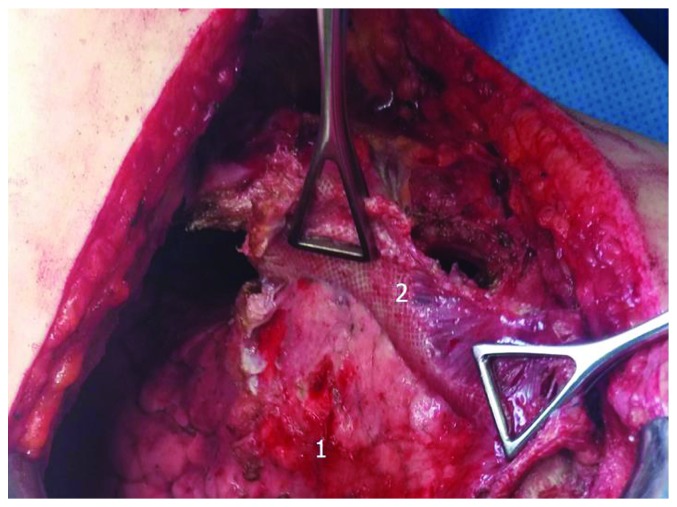
Separation of the mesh from the lung. 1: lung; 2: mesh.

**Figure 7:**
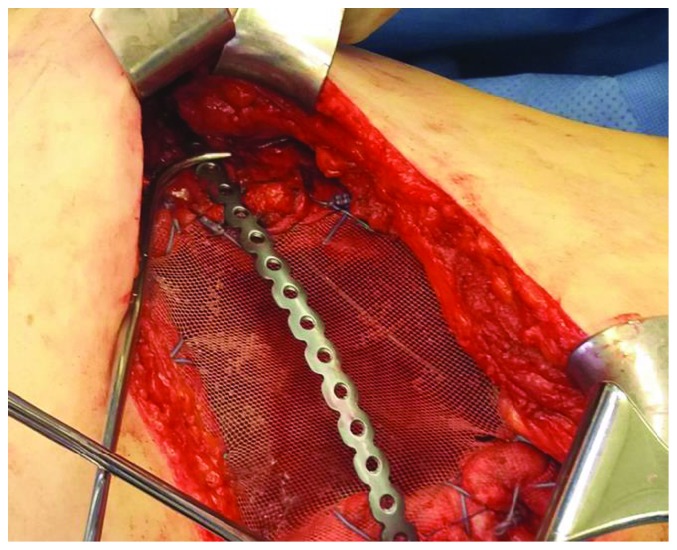
One Synthes plate fixated over the Mersilene mesh covering the chest wall defect.

**Figure 8:**
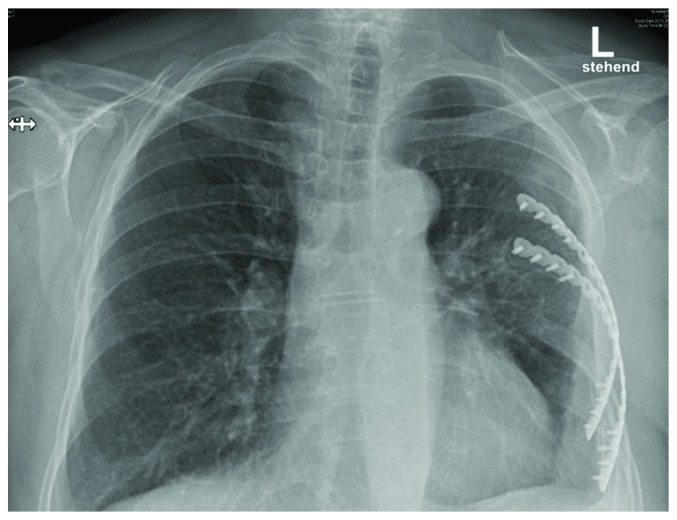
Chest radiography at discharge.

After the excision of the previous skin scar and the incision of the muscular layer, the mesh region was exposed, showing a lung protrusion (4 × 3 cm) along the anterolateral edge of the mesh (Figure 4). The local situation is schematically presented on Figure 5. The mesh suture line in the hernia region was completely disrupted, with a small piece of the herniated lung being completely detached from the mesh, the remaining lung surface under the mesh area being fully adherent to the mesh. By careful dissection, the mesh was separated from a firmly adherent lung and removed (Figure 6). After adhaesiolysis and complete lung liberation, a wedge resection of the afunctional lung tissue of the superior segment of the lingula was done, just in the region of contact with the mesh. After the chest tube insertion, the chest wall defect was reconstructed by suturing a Mersilene mesh in two layers—single pericostal sutures for initial fixation and running suture for additional reinforcement (Figure 7). A final chest wall stabilization was done by the fixation of two Synthes plates (DePuy Synthes J&J) over the 5th and 6th ribs (Figure 7). The postoperative course was uneventful. The chest X-ray on discharge, on postoperative day 5, is presented in Figure 8.

At the last contact with the patient, one year after the operation, the general condition was good, without the need for analgesics.

## 3. Discussion

The presented case can be of interest from at least three standpoints: (1) possible cause of the hernia; (2) interval between the hernia occurrence and diagnosis; and (3) hernia repair technique.

1. Postoperative lung hernias are usually associated with minimally invasive surgery, where the intercostal incision is longer than a skin incision [3]. It was not the case with the presented patient. On the other hand, it was also documented that chest wall defects alone are not sufficient to produce lung herniation. As a primary mesh interposition has been done in the presented patient, the defect size itself is less likely to have been a dominant factor causing lung hernia.
2. A mesh disruption in the anterolateral part and the technique of the mesh fixation (interrupted stitches) suggest that the upfront mesh reinforcement ought to have been done at a time of the first surgery. The patient reported episodes of dry cough preceding the hernia symptoms, a probable key factor causing mesh disruption.
3. According to the literature, most of the postoperative lung hernias occur weeks to months after surgery, like in the presented case. The probable explanation is, as previously documented, that signs of lung hernia, in the form of bulging with cough or palpable chest wall defects, may or may not be associated with symptoms, sometimes being even misleading. Treatment for an acute systolic heart failure or urgent intubation and embolisation for hemoptysis have been reported as well [4, 5]. In the presented patient, although the diagnosis of the hernia was obtained four months after surgery, the interval between the two operations was almost two years. The fact that the patient was not in favor of immediate hernia repair (only slight limitation in daily activities) supports some convincing literature data that although the hernia may not resolve it can dramatically improve, unless the patient is in distress [6]. In the presented case, our decision to perform such an operation was influenced by the opinion of radiologists that the CT aspect could be also suggestive of sarcoma. The decision to operate was also prompted by severe pains, being clearly increasing in intensity in comparison to the time of diagnosis.
4. From the standpoint of surgical technique, two points are to be mentioned. First, having in mind the localization of the defect, was a primary mesh reconstruction necessary during the first surgery and would the upfront stabilization have been better? The primary reconstruction was done during the first surgery because of the need to remove the scapula tip and to achieve stability with the time. The upfront stabilization was not deemed as necessary because the defect was not too big (including only two ribs) to justify the presence of the metallic foreign body. We do not recommend the support by metallic plates as the solution in general at initial surgery but only in case of correction failure. In case of anterolateral chest wall defect, we think that the mesh reinforcement should be taken into consideration as well.
5. Second, was titanium plate stabilization necessary and what would have been the alternatives? The chest wall rigidity restoration by using polytetrafluoroethylene, polypropylene, or composite meshes as well as methyl methacrylate sandwich is well established and sufficiently documented, including our earlier experience [7, 8]. In the presented case, we wanted to avoid methyl methacrylate sandwich in this redo surgery because of a quite high reported complication rate related to this technique.

Similar approach was described by using laminar hooks connected to titanium bars in one patient [9] and intramedullary titanium implants in two patients [10]. In the latter report, surgery was done for spontaneous hernia, with rib fractures in both patients with formation of the hernia ring. The reason for plate implantation was rib fractures, not the defect size. In the first report, this technique was used for the correction of spinal injuries and scoliosis. Apart from the obvious need to prevent hernia recurrence, our decision to use titanium plates was prompted by our earlier experience and some recent literature data suggesting clear functional benefits from such a technique [11–15]. A similar approach, but for completely different herniation type and smaller defect size, was reported by Bille et al. with two patients operated on for lung herniation after spontaneous rib fracture from severe bouts of cough [16].

No need for analgesics and good function of the shoulder girdle after the one-year follow-up demonstrate the appropriateness of the described technique.

To summarize, we believe that the hernia was caused by the tearing of the insufficiently thick soft tissue in the anterolateral intercostal area, where the mesh-fixating sutures were placed. As the mesh was initially fixated by interrupted sutures, we assume that an additional running suture (as performed during the redo surgery) would be helpful to prevent hernia occurrence. Based on our good experience with single sutures for mesh fixation, we do not suggest the upfront switch to running suture for the first suture layer. Furthermore, we do not suggest the upfront chest wall stabilization for hernia prevention. We suggest it only as a method of correction in case of failure of the initial postoperative hernia repair.
